# Identifying spatio-temporal dynamics of Ebola in Sierra Leone using virus genomes

**DOI:** 10.1098/rsif.2017.0583

**Published:** 2017-11-29

**Authors:** Kyle B. Gustafson, Joshua L. Proctor

**Affiliations:** Institute for Disease Modeling, Bellevue, WA 98005, USA

**Keywords:** Ebola, phylodynamics, disease modelling, spatial epidemiology

## Abstract

Containing the recent West African outbreak of Ebola virus (EBOV) required the deployment of substantial global resources. Despite recent progress in analysing and modelling EBOV epidemiological data, a complete characterization of the spatio-temporal spread of Ebola cases remains a challenge. In this work, we offer a novel perspective on the EBOV epidemic in Sierra Leone that uses individual virus genome sequences to inform population-level, spatial models. Calibrated to phylogenetic linkages of virus genomes, these spatial models provide unique insight into the *disease mobility* of EBOV in Sierra Leone without the need for human mobility data. Consistent with other investigations, our results show that the spread of EBOV during the beginning and middle portions of the epidemic strongly depended on the size of and distance between populations. Our phylodynamic analysis also revealed a change in model preference towards a spatial model with power-law characteristics in the latter portion of the epidemic, correlated with the timing of major intervention campaigns. More generally, we believe this framework, pairing molecular diagnostics with a dynamic model selection procedure, has the potential to be a powerful forecasting tool along with offering operationally relevant guidance for surveillance and sampling strategies during an epidemic.

## 1. Introduction

Arresting the West African Ebola virus (EBOV) epidemic of 2013–2016 required a significant international intervention and exposed a global vulnerability to emerging epidemics. Advances in genetic-sequencing technologies have enabled the near real-time analysis of infectious pathogen genomes [1–3], thereby improving forecasts for emerging epidemics [4,5], enhancing surveillance of endemic diseases [6] and identifying strategies for eradication [7,8]. During the West African EBOV crisis, publicly available data facilitated a series of prominent analyses aimed at identifying basic epidemiological parameters, i.e. the reproductive number [9,10]. Further, the release of EBOV genome data, coupled with phylogenetic methods, provided fundamental insight into the origin and spatial properties of the epidemic [11,12]. Despite the prominent role mathematical and statistical modelling played during the epidemic, there has been a significant delay in characterizing the spatio-temporal spread of EBOV. For future epidemics, the design of operationally relevant, spatially distributed interventions requires the identification of predictive models that are able to assimilate case and genetic data. In this article, we describe how EBOV molecular data, specifically virus genomes, can be used to directly model the spatio-temporal dynamics of the Sierra Leone epidemic.

Recent investigations of disease propagation on modern transportation networks have pointed to the importance of characterizing the spatial behaviour of vectors and pathogens due to human movements [13,14]. An influential development in the study of human mobility and disease spread is the adoption of the *gravity model* from the field of economics [15]. Analogous to the attracting force between physical masses, the gravity model describes human movements as dependent on the size of and distance between human populations [16,17]. Other spatial models, such as the well-known, scale-free Lévy flights, depend solely on travelling distance and have been used to describe a wide-ranging set of phenomena from epidemiology [18], ecology [19] and plasma physics [20].

Mathematically, the gravity and Lévy flight models are closely related. However, when the model parameters are fit to country-specific data, their dynamic behaviour can be qualitatively different. The gravity model parameters are often fit using proxy data such as cell phone, transportation and individual survey records [21]. This spatial model can then be coupled to a disease transmission model [22]. Alternatively, molecular data offer direct insight into disease mobility [4,23]. Genomic data have been used to construct distance-dependent spatial models for West Nile virus in North America [2] as well as dengue virus in Thailand [24] and Vietnam [25]. Other phylodynamic approaches, focused on mapping transmission trees [26], have been widely applied to infectious disease data including outbreaks of foot-and-mouth disease [27], severe acute respiratory syndrome (SARS) [28] and tuberculosis [29].

Previous spatial analyses and modelling efforts for the EBOV epidemic have identified population as an influential factor using a generalized gravity model parametrized to case-reporting data [30–32]. A comprehensive phylodynamic analysis of all available West African EBOV genomes also concluded that population distribution and distance between cases are important explanatory factors [33]. Other phylodynamic analyses of EBOV incorporated multiple countries and revealed the importance of social clustering to transmission risk [34,35]. Despite these recent investigations, which identify potential drivers of the EBOV epidemic, a fully characterized understanding of the spread of the West African epidemic remains a challenge.

We present a novel investigation of the EBOV genome data, allowing for a more resolved characterization of the spatio-temporal dynamics during the epidemic. Transmission of EBOV within Sierra Leone was almost completely within its borders [36], which provided a constrained and representative dataset to investigate the utility of virus genomes to construct population-level spatial models. Paired with advances in phylogenetics that identify linkages between cases [8], EBOV genome data offer powerful insight into spatio-temporal, transmission events. These genomic linkages, in combination with geographical and demographical characteristics included in our framework, help infer the parameters of gravity and Lévy flight models. We focus on identifying data-driven, spatial models that are interpretable and consistent with established patterns of human population movement. Adaptive model selection during the course of the epidemic reveals a significant change in virus mobility in Sierra Leone: dependence on population size decreases towards the end of the epidemic. For future epidemics, we believe that this framework could be implemented to improve forecasting efforts and help design efficient intervention campaigns that adapt to real-time phylodynamics.

## 2. Study data and methods

### 2.1. Genomic data

**Figure 1.**
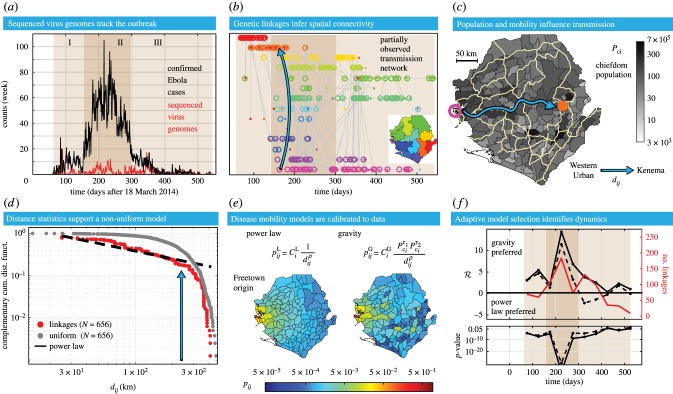
Virus genome data from EBOV cases in Sierra Leone characterizes the spatial spread of the epidemic. (*a*) The time course is shown for the number of confirmed cases [40] and sequenced EBOV genomes [33]. Three stages of the epidemic are highlighted. (*b*) Genetic linkages are illustrated with ancestors (open circles) and descendants (closed dots), both coloured by the origin district shown in the map key. The blue arrow highlights a linkage from the Western Urban to Kenema districts. (*c*) Chiefdom populations (greyscale) and major roads (yellow traces) are illustrated on the map of Sierra Leone. The blue arrow highlights the fastest driving route between the Western Urban to Kenema district. (*d*) All transmission distances are shown in a cCDF. The distribution of transmission distances are fit by a power law with *ρ* = 1.66. The blue arrow follows the linkage from (*b*) and (*c*). (*e*) Two spatial models are plotted as maps representing the probability of observing a new case linked to the Western Urban district, using (*ρ*^⋆^ = 1, *τ*^⋆^_2_ = 1) for the gravity model and *ρ* = 1.66 for the power law. (*f*) The log-likelihood ratio, 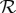, comparing the gravity and power-law models, is plotted for 50-day windows. The dashed black line represents (*ρ* = 1.66, *τ*_2_ = 1) fixed in time; the solid black line of 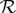 uses the MLE (*ρ*^⋆^(*t*), *τ*^⋆^_2_(*t*)), computed for each window. The solid red trace describes the number of linkages.

Genetic sequences from 1031 human infections of EBOV in Sierra Leone were obtained from a openly accessible compilation [33] of previously published sequencing data [36–39]. In figure 1*a* and electronic supplementary material, figure S4, we show the time course of all confirmed EBOV cases (black trace) in Sierra Leone [40] compared with the number of sequenced virus genomes [33] (red trace). The FASTA file with the genomes and metadata was downloaded from http://github.com/ebov/space-time/tree/master/Data/Makona_1610_genomes_2016-06-23.fasta on 9 August 2016. We then used BEAUTI 1.8.3 [41] with default options to generate an XML file with the metadata of spatial and temporal coordinates for each sequence.

### 2.2. Partially observed transmission network

We used a recently developed phylogenetic method [8], known as the partially observed transmission network (POTN) algorithm, to determine genetic linkages between EBOV infections in Sierra Leone. The POTN algorithm computes a likelihood ratio based on a Poisson model of the mutation rate to identify genomes that are most likely to be direct relatives. This contrasts with widely used phylogenetic analyses that infer common ancestors, such as Bayesian Evolutionary Analysis Sampling Trees (BEAST) [41]. The POTN algorithm produces a pairwise, time-directed network of ancestor and descendant genomes, linked by the relative change in their sequences between collection dates. For EBOV, we used an average nucleotide substitution rate of 2 × 10^−3^ bp/site/yr, a value measured during the 2013–2016 epidemic; see Fig. 4F of [11]. A false discovery rate for each linkage is computed with a single degree-of-freedom *χ*^2^ test, with a cut-off at *p* = 0.05. Figure 1*b* shows a visualization of the EBOV POTN for Sierra Leone pruned to the shortest generation time for each ancestor. The blue arrow highlights a single linkage between virus genomes collected in the districts of Western Urban and Kenema.

### 2.3. Population distribution and driving distances

Population distribution maps from the 2010 and 2014 Worldpop models were downloaded from http://worldpop.org.uk/data/ in June 2016. These maps were segmented into 153 Admin-3 units (chiefdoms) using the Sierra Leone shapefiles from Global Administrative Areas http://gadm.org/download, illustrated in figure 1*c*. Driving distances were used as the distance measure between chiefdoms. The shortest time driving distances between all chiefdom pairs were collected from the Google Maps API. Figure 1*c* shows the major roads in Sierra Leone. The blue arrow indicates the path along the roads between chiefdoms of median population in the districts of Western Urban and Kenema.

### 2.4. Distance statistics for genetic linkages

We examined the statistics of EBOV transmissions using the distribution of driving distances between POTN-linked cases. These are denoted as transmission distances, *d*_*ij*_. We plot in figure 1*d* the probability of observing a *d*_*ij*_ above a certain magnitude, which is defined as a complementary cumulative distribution function (cCDF), and is useful for identifying a power-law distribution from empirical data [42]. We computed *d*_*ij*_ for each genetic linkage by assigning each sequence to a chiefdom, either known from the metadata or approximated by population size in its annotated district. We omitted 119 genomes without a district (Admin-2) localization from the analysis. For the first part of the epidemic, chiefdom localizations are available for 187 genomes [36]. When the chiefdom localization is unknown for a virus genome, we selected a chiefdom based on assumptions of population size within the known district, such as maximum, mean, median or minimum.

### 2.5. Probabilistic spatial models

Previous analyses have pointed to the importance of size and distance between populations as factors that influenced the spread of EBOV in West Africa [31,33]. Here, we specify a gravity model for a discrete spatial network of populations that describes the probability of a virus being transmitted from chiefdom *i* to chiefdom *j*: *p*^G^_*ij*_ = *C*^G^_*i*_*P*^*τ*_1_^_*i*_*P*^*τ*_2_^_*j*_/*d*^*ρ*^_*ij*_, where the origin population is *P*_*i*_, the destination population is *P*_*j*_ and *C*^G^_*i*_ normalizes the probability distribution for each origin. The exponents *τ*_1_, *τ*_2_ and *ρ* are parameters that determine the influence of population and distance for the gravity model. The normalization for each origin is computed by the following: 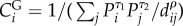. Note that the normalization depends solely on the destination population and distance. This formulation of the gravity model predicts where a future linked case will appear.

A closely related probabilistic model is the Lévy flight model, which has a rich mathematical basis in the framework of fractional diffusion equations and scale-free non-diffusive random processes [43]. We write the discrete space power-law model as *p*^L^_*ij*_ = *C*^L^_*i*_/*d*^*ρ*^_*ij*_, where *C*^L^_*i*_ normalizes the probability for each origin. Again, we are interested in characterizing the probability of viral transmission to chiefdom *j*. The resting probability for both models is uniformly approximated to *p*_*ii*_ = 0.5; see electronic supplementary material, figure S1 for a district-level analysis of stationary linkages. This approach can be extended to include a wide variety of spatial models with context-appropriate parameters for the underlying stochastic process.

### 2.6. Maximum-likelihood estimates for gravity model parameters

For the gravity model, the parameters *ρ* and *τ*_2_ that best fit the data can be determined through a maximum-likelihood estimate (MLE). The joint likelihood for the parametric gravity model, 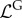, is defined as the product of model evaluations over the set of virus genome linkages 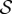: 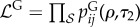. We define (*ρ*^⋆^, *τ*^⋆^_2_) as the MLE of the parameters for the gravity model determined by evaluating the likelihood for a range of (*ρ*, *τ*_2_) values. We establish a 95% CI for (*ρ*^⋆^, *τ*^⋆^_2_) via the well-known Fisher information criterion [44].

### 2.7. Adaptive model selection

We computed a time-dependent likelihood ratio that quantifies the relative preference between models over the course of the epidemic. Note that the power-law model is considered nested within the gravity model if *τ*_2_ → 0. The likelihood ratio, 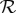, is computed for a set of virus genome linkages 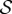. The normalized log-likelihood ratio of a gravity model to a Lévy flight model is 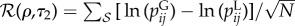, where *N* is the number of linkages in 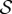. If 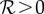, the gravity model is preferred, but if 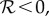 the power-law model is preferred. The significance of this preference is computed by a *χ*^2^ test according to Wilks' theorem [45]. We made 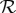 time-dependent by partitioning 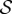 into subsets of linkages, 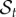. In figure 1*f* , each subset includes all linkages with the descendant genomes collected in each 50 day interval centred around *t*. This model selection approach can be extended to include non-nested models by using an information criterion such as the Akaike information criterion [46].

## 3. Results

### 3.1. A transmission network links most virus genomes

We constructed a POTN using 880 virus genomes from Sierra Leone that revealed 798 transmission events. Of these, 355 have a unique descendant and 670 have fewer than three likely descendants. The POTN algorithm is designed to return several possible descendants associated with the same ancestor when these are supported by the data. See electronic supplementary material, figure S2A for the empirical distribution of all linkage durations. For illustrative purposes, figure 1*b* shows the POTN pruned to include only the shortest linkage duration from each ancestor. The median linkage time from the POTN is 31 days. Based on an EBOV serial interval of 15 days [47], this implies approximately one unobserved transmission event per linkage. The median linkage duration is reduced to 11 days if the POTN is pruned to the shortest generation times, illustrated in electronic supplementary material, figure S2B.

The likelihood ratio and MLE calculations include all likely descendants associated with a single ancestor. However, for the adaptive model selection procedure, descendants outside of each time interval are excluded. For example, figure 1*f* illustrates the likelihood ratio for 50 day intervals. Despite the challenges associated with partially observed transmission chains, we find that our subsequent analyses of spatial model calibration and model selection are robust to a wide variety of linkage exclusion criteria, such as restricting the maximum allowable linkage duration. See electronic supplementary material, figure S3 for more details. Our results are also robust when considering longer time intervals. We define three sequential stages for the epidemic: Stage I (0–150 days), Stage II (150–300 days) and Stage III (300–550 days). The geographical distribution of linked cases across districts for the three stages shows that the number of genomes sequenced is proportional to the number of confirmed cases [40], except when the number of confirmed cases is larger than 1000 (electronic supplementary material, figure S4).

### 3.2. Transmission distances follow a power-law model

**Figure 2.**
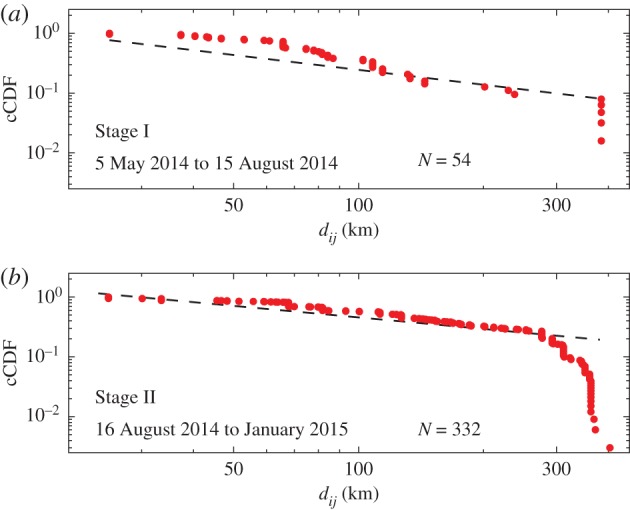
The empirical power law for the transmission distances. (*a*) The complementary cumulative distribution function (cCDF) for Stage I, (50 ≤ *t* < 200 days), is plotted along with the power-law model using the MLE value of ρ* = 1.8 ± 0.1 for *N* = 54 linkages. (*b*) The cCDF for Stage II, (200 ≤ *t* < 350 days), is plotted with the power-law model using the MLE value of *ρ* = 1.6 ± 0.1 for *N* = 332 linkages.

Several analytic techniques were used to test for a power law in the distribution of *d*_*ij*_ for all linkages. Cumulatively, for 656 linkages with *d*_*ij*_ > 0 km, we computed a power-law scaling exponent of *ρ* = 1.66 ± 0.02 for the discrete distribution of *d*_*ij*_, as shown in figure 1*d*. We also found that *ρ* is consistent across different stages of the epidemic, as shown in figure 2 and electronic supplementary material, figure S5. This estimate for *ρ* was computed using a well-known maximum-likelihood method for power-law distributions [42]. As a note of caution, the methodology in [42] provides a lower bound on the distance to define a power-law tail, whereas we have explicitly included all transmission distances here to remain unbiased. We verified that the power law is preferred by the likelihood ratio over a Weibull or exponential probability distribution.

As a separate investigation, a two-sample Kolmogorov–Smirnov test showed that the distribution of *d*_*ij*_ is not likely drawn from the same distribution as all the possible driving distances between chiefdoms. Therefore, the transmission events do not match a uniform random process on the driving network. We also examined the sensitivity of the model fit by sampling from the inferred model and driving distance distribution. By randomly drawing a similar number of samples from the inferred model and driving network, we found that the model fit is robust to the number of samples collected during the epidemic; see electronic supplementary material, figure S6 and accompanying text for more details. The observed distribution of *d*_*ij*_ is closely related to a power law for a significant portion of the data, shown in electronic supplementary material, figure S6.

However, there are clear differences between the simulation of the idealized power-law model and the distribution of *d*_*ij*_ that suggest the influence of other factors. For example, the geographical structure of Sierra Leone constrains the number of possible trips over 300 km. This is consistent with the observed drop-off in transmission distances, indicating a limitation of the standard power-law model for the complex effects of national borders and local administrative divisions. Further, including other factors, such as the chiefdom-level population distribution, will allow for more flexibility in characterizing the observed distribution of transmission distances.

### 3.3. Gravity model at epidemic peak was driven by Freetown

**Figure 3.**
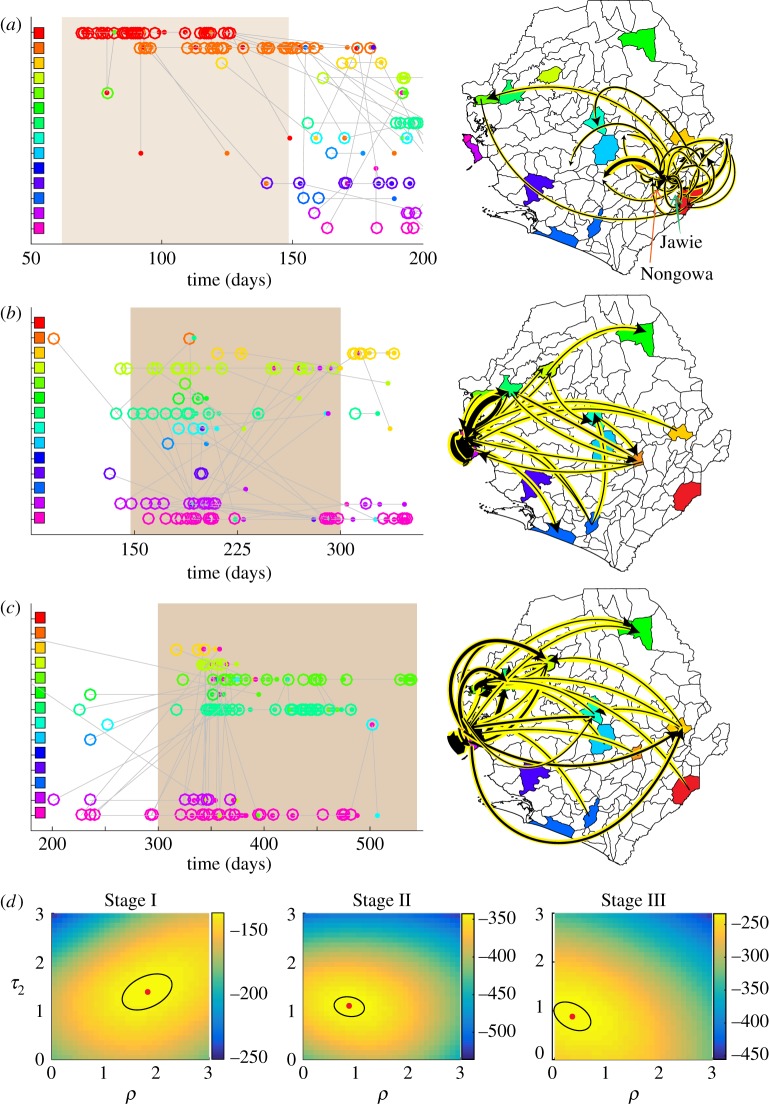
The partially observed transmission network (POTN) and estimation of parameters for the gravity model. (*a*)–(*c*) The left column illustrates the POTN for Stages I–III. The open circles and closed dots represent ancestors and descendants, respectively. Each are coloured by the ancestor district. Linkages of shortest duration are shown. The right column illustrates the POTN linkages on a map with black arrows highlighted in yellow. The black arrow width is proportional to the number of linkages. (*d*) The likelihood evaluation is illustrated for each stage along a grid of values (*ρ*, *τ*_2_). The MLE and 95% CI are illustrated by a red dot and black ellipse, respectively.

Inferring the parameters of the gravity model with the genetic linkage data, population was found to be an important variable in characterizing spatial transmission events, especially in Stage II of the epidemic when the Western Area is involved in 244 of the 363 genetic linkages. In Stage II, the MLE of the gravity model parameters found *τ*^⋆^_2_ = 1.2 ± 0.3 and *ρ*^⋆^ = 0.9 ± 0.5 with a 95% CI; see electronic supplementary material, figure S7 for more details on the MLE calculation. The likelihood landscapes, with varying (*ρ*, *τ*_2_), are shown in figure 3*d* for Stages I–III. Values of the log-likelihood for each stage and both models are shown in electronic supplementary material, figure S9. The likelihood ratio, comparing the gravity and power-law models during Stage II, indicated a strong preference for gravity shown in figure 1*f*. Further, figure 3*b* illustrates that the POTN for Stage II contains a significant number of transmission events in the Western Area of Sierra Leone supporting the population-dependent model. When setting the population parameter *τ*_2_ to the canonical value of 1, the gravity model was still preferred over the power law for this portion of the epidemic. Figure 1*e* illustrates gravity and power-law models as chiefdom-level maps of Sierra Leone with Freetown as the origin of a virus genome. The Stage II virus sequence data were consistent with a destination-population gravity model dominated by Freetown linkages.

### 3.4. Adaptive model selection identifies a change in dynamics

The likelihood ratio helped identify a changing preference of the gravity model over the course of the epidemic. Figure 1*f* illustrates this preference change with 50-day windows. Stage I exhibited a weaker preference for the gravity model than Stage II. The 119 sequences of Stage I came from the work of a single team [36] and included chiefdom localization linking 70% of cases in Stage I to either Jawie chiefdom in Kailahun district or Nongowa chiefdom in Kenema district, shown in figure 3*a*. Most of the linkages occurred in these larger population chiefdoms in the eastern province of Sierra Leone, illustrated on the map of figure 3*a*. The MLE estimate for the population parameter of the gravity model in Stage I found *τ*^⋆^_2_ = 1.5 ± 0.5; see electronic supplementary material, figure S7 for each stage. The likelihood landscape also shows a distinct shift to a stronger population dependence and smaller expected transmission distances than found in Stage II, shown in figure 3*d*.

The preference for the gravity model decreased substantially after 300 days. In Stage III, for 261 linkages with descendants in the final 250 days of recorded genomes, the MLE for the gravity model parameters found *τ*^⋆^_2_ = 0.8 ± 0.3 and *ρ*^⋆^ = 0.5 ± 0.5. However, the likelihood ratio revealed that the Stage III gravity model was not significantly preferred over the power law whether using the MLE of (*ρ*^⋆^, *τ*^⋆^_2_) or the canonical gravity model. Further, a power law model was *weakly preferred* after February 2015 when considering shorter 50-day windows and setting the population parameter *τ*_2_ = 1, as shown in figure 1*f* . From the Stage III map in figure 3*c*, we note that a more diverse scattering of linkages across Sierra Leone supports a qualitative change in the dynamic behaviour of the epidemic.

### 3.5. Sensitivity analysis of missing chiefdom data

The results in this article are largely consistent regardless of the chiefdoms assigned to genomes with only district-level localization. For sequences with unknown chiefdoms, we selected the median population chiefdom from the known district. Electronic supplementary material, figure S8 illustrates how the likelihood ratio trajectory over the course of the epidemic depended on this assumption. A similar qualitative trend is identified whether choosing the maximum, minimum, median or mean population chiefdom. However, the identified statistical change in model preference from gravity to power law after February 2015 was sensitive to this assumption, especially when using the maximum population chiefdom for each district. In Fang *et al.* [40], a majority of confirmed cases have chiefdom annotations except in the Western Area. Both the confirmed cases and virus genomes recorded during Stage I indicate that most cases in the Kenema district are from highly populated chiefdoms. However, in Stage III, most confirmed cases are in chiefdoms closer to the median population; see electronic supplementary material, table S2 for more details.

## 4. Discussion

Understanding the changing spatio-temporal dynamics of an emerging epidemic is fundamental to designing real-time disease interventions. Data, gathered from case-contact tracing and molecular diagnostics, can identify individual transmission events that inform population-level models of disease spread. For example, analyses of recent epidemics, including EBOV outbreak in West Africa [31,33,48], the SARS outbreak in 2003 [49] and Middle East Respiratory Syndrome (MERS) outbreaks in 2012 [50], each used detailed individual-level data to infer epidemic parameters and factors influencing large-scale dynamics. Despite the encouraging progress of mathematical modelling and statistical analyses for the 2013–2016 EBOV epidemic [10,33,51,52], the characterization and spatial modelling of the outbreak is incomplete. The ability to rapidly quantify spatio-temporal spread *during* an epidemic would allow for near real-time forecasts and the design of operationally relevant, spatially targeted interventions. The primary contribution of this work is the development of an adaptive framework for analysing epidemics that incorporates detailed transmission information from linked virus genomes to characterize interpretable, population-level spatial models.

Recent advances in phylogenetic reconstruction of transmission networks promise accurate and actionable models of epidemic dynamics [4,27–29,53,54]. Here, we chose the POTN method [8] as an efficient and direct likelihood-based tool to link EBOV cases in space and time. These high-fidelity, space–time couplings between individual cases allowed the parametrization of spatial models describing *disease mobility*, without the need for proxy human mobility data. This framework offers a principled and extensible methodology for investigating the relevant factors for disease mobility.

Our results are largely consistent with other investigations of the spatio-temporal spread of the EBOV epidemic. Previous spatial modelling, with or without virus genome sequences, has concluded that distance, population density and international border closures are covariates that help predict the probability of transmission [31,33]. Other modelling studies have indicated that large population centres, such as Kenema and Port Loko in Sierra Leone, are responsible for initiating self-sustaining local outbreaks [55]. In our investigation, we confirmed that a population-dependent model is preferred when aggregating all transmission events during the epidemic [33].

We have broadened the scope of previous analyses by identifying how the influences of population and distance on the spread of EBOV *change* over the course of an epidemic. A wide variety of probabilistic models can be proposed to describe the stochastic spatial process underlying disease transmission during an epidemic. For this study, we posited two parsimonious models, well known in the epidemiology and ecology literature, to investigate the influence of population and distance on the spatial spread of cases. We discovered that the stochastic propagation of cases is best described by a probabilistic gravity model where dependence on the population and distance varies over the course of the epidemic. The gravity model was preferred in the early part of the epidemic when EBOV was circulating near cities in the east of Sierra Leone. Once the virus migrated to more densely populated areas of the capital area, such as Freetown, Kenema and Port Loko, the gravity model preference became much stronger. During this portion of the epidemic, the transmission events were highly local with a large proportion of linkages staying between large population centres. This observation is also consistent with recent studies of the superspreader phenomenon in the Western Area [52,56].

The probabilistic gravity model can be considered a generalization of a random walk process, weighted by country-specific population distributions. This population influence changed over the course of the EBOV epidemic. In fact, after March 2015, the population dependence diminished significantly. This suggests that EBOV mobility in the last stage of the epidemic can be accurately modelled as a spatial process dependent solely on distance. The MLE of the parameters for the gravity model showed a large uncertainty in the population exponent *τ*_2_. Further, the distance exponent was *ρ* < 1, indicating a higher probability of larger distances between linked cases. In the pure power-law model, the disease mobility during this period has a Lévy flight exponent of order *α* = *ρ* − 1 = 0.6, suggesting a space-fractional diffusion process. This result is consistent with the observation that confirmed EBOV cases decreased in the large cities of the Western Area and appeared sporadically in less populated areas far from the Western Area after March 2015. Further, this shift away from a strong preference for a gravity model coincided with an intervention campaign by the government of Sierra Leone called *Operation Western Area Surge* (OWAS) that occurred on 17 December 2014 [57]. Sociological observations, after the OWAS, described an increase in health centre avoidance, return trips to home villages and transmission away from population centres [58]. These results highlight the importance of continual collection of genomic data for characterizing the change in dynamic behaviour along with evaluating the effectiveness of interventions.

Surveillance difficulties during an epidemic pose constraints on our framework being used as a forecasting tool. Despite the EBOV data spanning the entire country and nearly the full time course of the epidemic, the collection of virus genomes was not part of a unified programme. Moreover, the metadata for each sequence, i.e. the global positioning system location and demographical information, are not completely resolved. Uncertainty in reporting due to collection and laboratory processing introduces delays that could impact the utility of predictive spatial models. Our model selection also currently consists of two classes of spatial models: gravity and Lévy flight. We expect to expand our framework to a wider variety of models, but are aware of the challenge in finding parsimonious descriptions of human mobility [59]. As a retrospective study, we have analysed the robustness of our methodology to uncertainties, but inherent difficulties in data collection and modelling will challenge real-time deployment of this tool.

Notwithstanding these limitations, our study can provide operational guidance into the number of collected virus genomes and acceptable time frames required to inform spatial models for prediction. Our model selection technique showed that virus genomes can potentially help characterize the impact of intervention campaigns during an epidemic. Looking towards the next emergence of a dangerous pathogen, molecular diagnostics paired with dynamic models are poised to become a new benchmark for uncovering epidemiological patterns [6], forecasting disease propagation [5] and informing interventions [60] for a wide variety of infectious diseases.

## Supplementary Material

Supplemental Information

## Data accessibility

We have made the source code and data files for our analysis available at https://github.com/kgustafIDM/disease-mobility.
