# Supplementary material for: Identifying spatio-temporal dynamics of Ebola in Sierra Leone using virus genomes
Source: J R Soc Interface. 2017 Nov 29;14(136):20170583. doi: 10.1098/rsif.2017.0583 (PMC5721162; doi:10.1098/rsif.2017.0583)
Supplement: Supplemental Information [file rsif20170583supp1.pdf]

Identifying spatiotemporal dynamics of Ebola in Sierra Leone using virus genomes

Kyle B. Gustafson and Joshua L. Proctor

SUPPLEMENTAL INFORMATION

We have made the source code and data files for our analysis available at `github.com/kgustafIDM/disease-mobility`.

A. Population statistics

The populations of chiefdoms range across Sierra Leone from 2924 (Toli, Kono district) to 699584 (central Freetown), with a median value of 25000 people. Freetown is split into five chiefdoms while other major cities are included in the nearest chiefdom. There are four large cities that are included in their nearest chiefdoms: Bo (pop. 211000, Kakua chiefdom in Bo district), Makeni (pop. 105000, Makari Gbanti chiefdom in Bombali district), Kenema (pop. 214000, Nongowa chiefdom in Kenema district), and Koidu (pop. 194000, Gbense chiefdom in Kono district).

B. Probability of a stationary linkage

The discrete gravity and power law model are not defined when $d_{ij} = 0$. However, there are a significant number of stationary linkages, where both the ancestor and descendant pair are in the same chiefdom. If the district (Admin-2) localization is the highest available resolution, we assume the linkage remained in the same chiefdom. We assigned a uniform empirical value of $p_{ii} = 0.5$ to approximate the average fraction of stationary linkages that remain in the same chiefdom. We investigated the sensitivity to this modeling choice by varying this stationary probability from 0.3 to 0.7. We did not find a significant difference in the likelihood ratio calculation. The empirical fraction of $d_{ij} = 0$ and the number of $d_{ij} = 0$ linkages are shown across all districts in Figure S1.

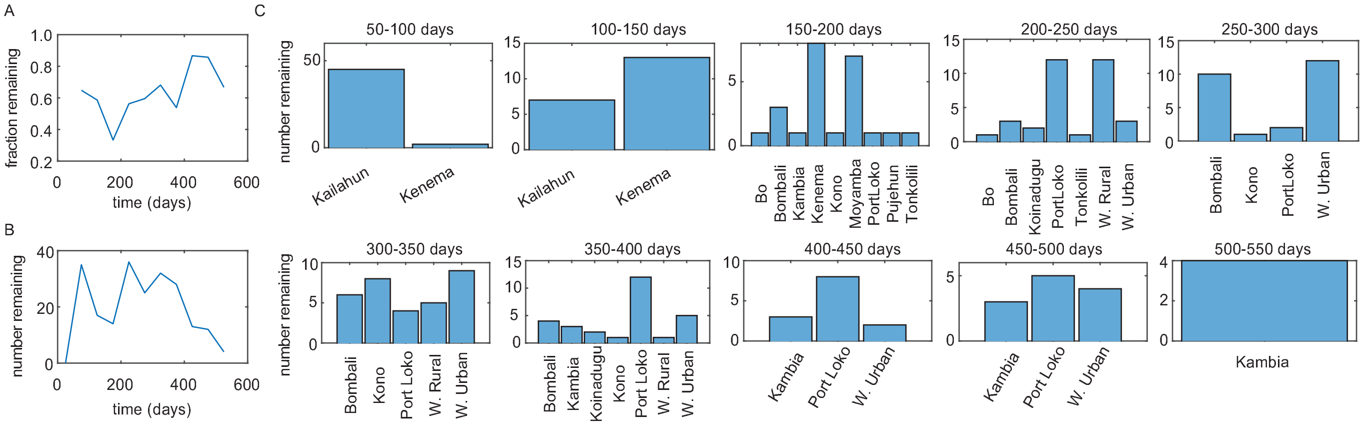

FIG. S1. Number and fraction of stationary linkages. (A) The fraction of stationary linkages across all districts is shown in 50 day windows. (B) The number of stationary linkages across all districts is shown in 50 day windows. (C) The number of stationary linkages in each district is shown in 50 day windows.

C. Distribution of linkage durations

The distribution of time delays between linked cases, denoted as linkage durations, is shown in Figure S2(A). The linkage durations have a median (mean) value of 31 (46) days with a maximum of roughly 250 days. However, the serial interval for EBOV infections was reported to be roughly 14-15 days[47]. The difference in the serial interval and the mean of the distribution of linkage durations arises from the fact that there are unobserved transmissions. The partially-observed transmission network (POTN) algorithm, will return multiple descendants to a single ancestor if the data suggests a similar likelihood. For example, the distribution of linkages times from the POTN can be pruned

to include only the shortest linkage duration for each ancestor, shown in Figure. S2(B). The observed median (mean) linkage duration in this case is reduced to 11 (27) days, though there remains a long tail in the distribution. We also note that there are 64 linkages duration of 0 days, which are likely to be associated with closely linked cases that have errors in their reporting dates. This is consistent with 56 of these linkages remaining in the same district or chiefdom, when the chiefdom was known. The remaining eight linkages with zero day duration may be due to reporting errors, however, our results are robust to excluding these linkages.

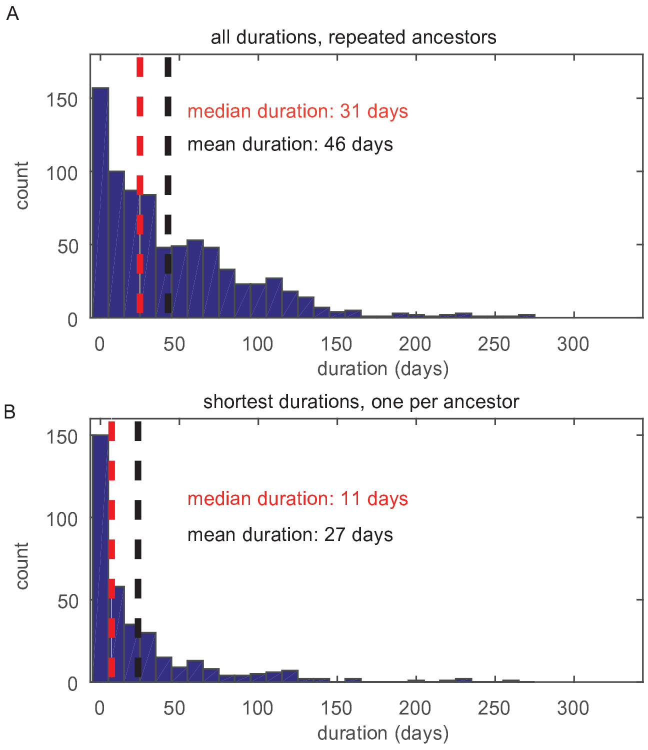

FIG. S2. Distribution of linkage durations (A) The empirical distribution of linkage duration times are illustrated for all ancestor-descendant pairs. (B) The empirical distribution of duration times are illustrated using the shortest linkage duration for each ancestor.

D. Sensitivity of the likelihood ratio to the inclusion of long duration linkages

We examined the sensitivity of our likelihood ratio calculation by excluding long duration linkages. In Figure S3(A), the likelihood ratio is plotted for: all linkages, only the shortest linkages for each ancestor-descendant pair, a 30-day maximum for linkage duration, and 60-day maximum for duration. Panel (A) kept the gravity model parameters fixed during the time-course at values ($\rho = 1.6, \tau_2 = 1$). Qualitatively, all of the likelihood traces under these assumptions are similar. Figure S3(B) illustrates a similar calculation, but the gravity model parameters were set according to their MLE values ($\rho^\star, \tau_2^\star$) computed in each 50-day window. In general, the same trends for the likelihood ratio regardless of upper bound on linkage duration are observed. When the linkages are pruned to the shortest durations, the likelihood ratio ceases to be statistically significant ($p = 0.05$) at $t = 350$ days when using $\rho = 1.6$ and $\tau_2 = 1$. However, the pruned result remains significant until $t = 425$ days when using the MLE for the gravity parameters for each window. The loss of significance in the p-value always occurs when fewer than 50 linkages are observed in a time window. This suggests a number of genomes that need to be collected during an epidemic to calibrate these spatial models with statistical significance for the desired time window.

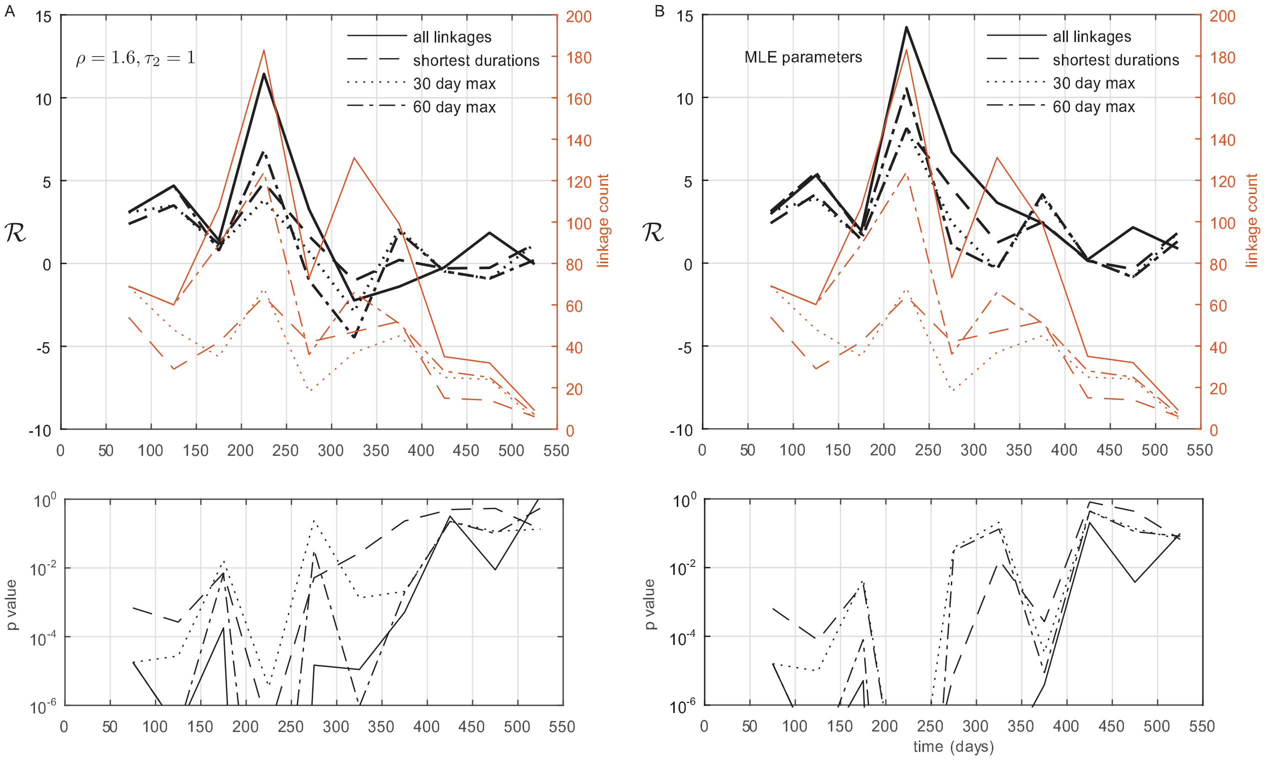

FIG. S3. Examining the sensitivity of log-likelihood ratio to linkage pruning. The likelihood ratio is plotted for all linkages, the shortest ancestor-descendant pairs, 30 day maximum for durations, and 60 day maximum for durations. (A) The likelihood ratio is plotted for fixed gravity model parameters ($\rho = 1.6, \tau_2 = 1$) for the entire epidemic. (B) The likelihood ratio is plotted similar to (A), however, the gravity model parameters were set according to their MLE values ($\rho^\star, \tau_2^\star$) computed in each 50-day window.

TABLE S1. Sequenced genomes and confirmed cases. For each district, the number of sequenced genomes[33] to confirmed cases[37] is tabulated for the three epidemic stages. Each stage is indicated with the center of its time window.

days	W. Urban	W. Rural	Port Loko	Kambia	Moyamba	Bonthe	Bombali	Tonkolili	Koinadugu	Bo	Pujehun	Kenema	Kono	Kailahun
425	81/371	24/97	89/190	63/113	0/7	0/0	53/51	4/12	4/6	53/51	0/0	1/3	31/20	0/1
225	71/1878	64/1037	59/975	10/126	21/192	1/5	52/990	14/473	7/105	61/1274	9/28	24/221	7/235	2/201
75	0/12	0/6	1/26	1/1	2/8	0/1	0/7	1/3	0/0	5/43	0/3	46/273	1/8	99/332

E. Sampling of genomes compared to confirmed cases

We had access to both the summary of sequenced EBOV genomes[33] and confirmed cases of EBOV infection[37] from Sierra Leone. We compared the number of sequenced and confirmed cases for each district by stage of the epidemic (0-150 days, 150-300 days, and 300-550 days). A summary of this comparison is shown in Figure S4 and Table S1. The area of each red circle represents the number of sequenced genomes whereas the area of each black circle represents the number of confirmed cases. For some districts, nearly all the cases were sequenced. In Bo and Bombali districts during the last stage, more cases were sequenced than confirmed. The extra Bombali sequence is due to an earlier cutoff in the confirmed case dates. The extra Bo sequence may be due to a difference in collection protocol and labeling. Overall, the sequenced cases track the confirmed cases proportionally across districts. However, there is a saturation in the number of sequenced genomes in stage II. No more than 100 genomes were sequenced in any single district. The difference between sequenced and confirmed cases was largest when nearly 2000 cases were confirmed in Western Urban district. However, we note that this does not change the qualitative conclusion for the preference of the gravity model in stage II. In fact, if more genomes had been sequenced in the Western Urban, the gravity model would likely be even more strongly preferred.

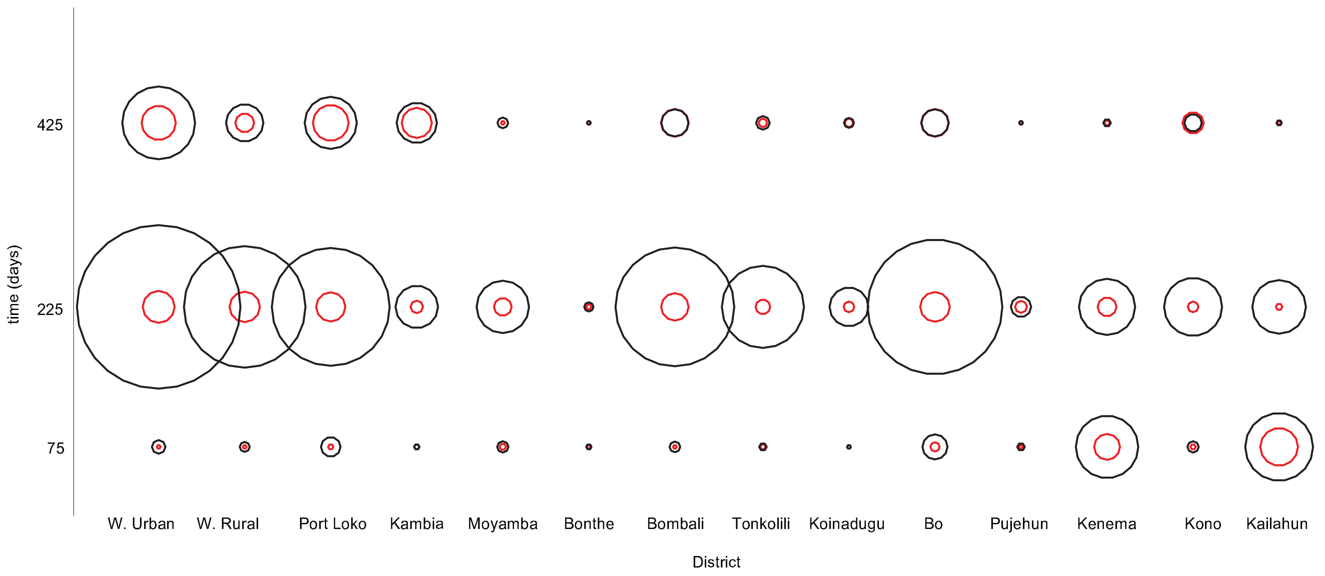

FIG. S4. Sequenced genomes and confirmed cases. For each district and epidemic stage, the number of confirmed cases is represented by a black circle. The number of sequenced genomes is represented by a red circle. Each stage is indicated by the center of its time window noted on the y-axis. The area of each circle is proportional to the number of cases at each point in time and geography. All counts are included in Table S1. The smallest circles in this visualization, in Bonthe during stage I for example, indicate zero observed cases.

F. Power law spatial model for transmission distances

We examined the statistical properties of a power law fitted to the distribution of driving distances, d_{ij}, that connect genetic linkages for EBOV disease cases in Sierra Leone. As a companion to Figure 2, the power law fit for stage III is shown in Figure S5. We also compared the power law fit to other common probability models such as the Weibull and stretched exponential, finding that the power law is a better fit for most of the range of d_{ij}. Additionally, we simulated a uniform distribution by aggregating driving distances between all pairs of chiefdoms, as shown in Figure S6. The distribution of d_{ij} is closer to a power law than a uniform distribution according to the two-sample Kolmogorov-Smirnov statistic. This statistic, D_n, measures the distance between two probability distributions, such that $D_n = 0$ for equivalent distributions. The value of D_n is $10 - 25\%$ smaller for the power law than for the uniform distribution across 1000 subsamples of $N = 656$ driving distances. We included all of the distances $d_{ij} > 0$ km in contrast to the standard practice of defining a lower bound on the power law fit[42]. The preference for a power law is observed despite the tail, $d_{ij} > 300$ km, of the distribution being closer to the uniform distribution than a power law.

While it is common practice to set upper and lower bounds for empirical identification of power law tails[42], we believe it is more operationally relevant to include all chiefdom pairs in our method. When distances are measured on a discrete, politically-defined topology, there are complex effects on the distance distribution due to national borders and heterogeneity in the size of administrative divisions. An important future research direction is investigating interpretable bounds on an empirical distance distribution for models defined on realistic spatial topologies. For example, the gravity model has been modified[21] to allow for piecewise assortative mixing based on distances.

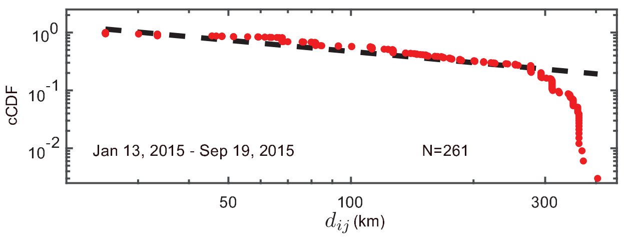

FIG. S5. Power law for transmission distances with all linkages in stage III. The complementary cumulative distribution function for transmission distances d_{ij} is shown for linkages in stage III ($350 \leq t < 500$ days) of the epidemic showing the maximum likelihood value of $\rho^\star = 1.7 \pm 0.1$ for 261 non-stationary linkages.

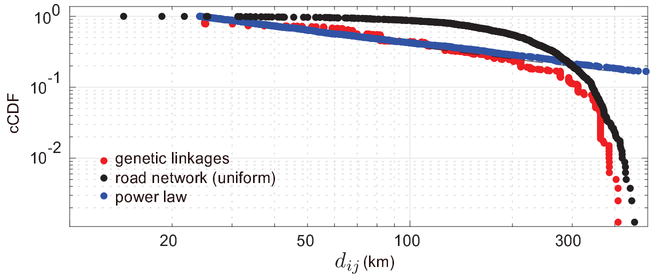

FIG. S6. Numerical simulations of an idealized power law and uniform driving distances. The complementary cumulative distribution function is shown for d_{ij} (red) and two distributions for comparison: a power-law distribution (blue) and uniform driving distances between chiefdoms in Sierra Leone (black). For each model, the same number ($N = 656$) of random draws were taken from the model distributions as for the transmission distances.

G. Maximum likelihood and confidence intervals for gravity model

Over the course of the epidemic, we computed the MLE for the parameters of our destination-population gravity model. The uncertainty of the MLE values can be computed using the observed Fisher information. The curvature of the likelihood surface at the MLE of the parameters helps define the confidence intervals. We show the time-course of these estimates with 95% confidence intervals for the three stages of the epidemic in Figure S7. We see both MLE values shrink, indicating a drop in the importance of chiefdom population size and an increase in the relative number of long d_{ij}. Since the uncertainty in these parameters is fairly large, we also studied the results of fixed parameter values ($\rho = 1.6, \tau_2 = 1$) for the gravity model. This allowed us to analyze the importance of population size while holding ρ at the level determined by the pure power law fit.

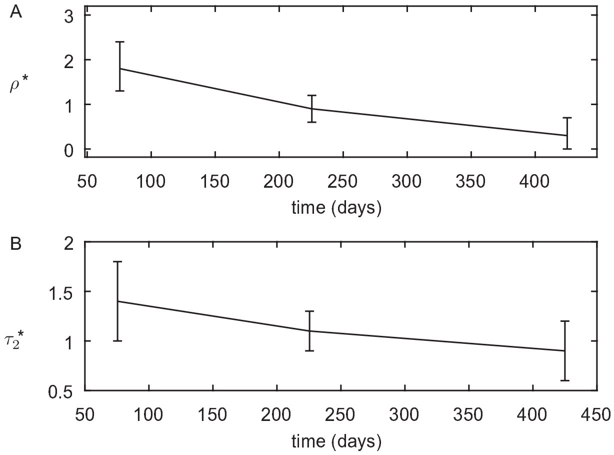

FIG. S7. The MLE for the gravity model in each stage of the epidemic. (A) The MLE, $\rho^\star$, is plotted for stages I-III. The 95% confidence interval is shown, calculated using the observed Fisher information. (B) The MLE, $\tau_2^\star$, is plotted for stages I-III. The 95% confidence intervals are also shown.

H. Sensitivity to unknown chiefdom localization

When chiefdom localization for cases was unavailable, we assigned each case to the median populated chiefdom in the known district. If a sequence had neither chiefdom nor district information, it was excluded from the analysis. We examined the sensitivity of our results to this assumption by recomputing the likelihood ratio for the minimum, mean, and maximum chiefdom population. Figure S8 illustrates the likelihood ratio for each of the population assumptions. We observed the same general trend in likelihood ratio: the gravity model is strongly preferred at the peak of the epidemic; the preference for the gravity model quickly fades toward the latter part of the epidemic after Operation Western Area Surge. Before 125 days, most chiefdoms are known in the data so the various assumptions do not change the result. However, there are subtle differences in the second and third stages of the epidemic. Comparing the minimum and maximum population, the power law model is statistically preferred for a longer period of the epidemic when using the minimum population. Conversely, the maximum population shows the gravity model strongly preferred for the entire epidemic. Note that this is consistent with our understanding of the probabilistic gravity and power law model. By choosing the maximum population chiefdom, we are biasing the result toward a population dependent model. Conversely, by choosing the minimum, we are biasing the result away from gravity and toward the distance-dependent power-law model. Importantly though, the qualitative trends in the likelihood ratio are robust to these population assumptions.

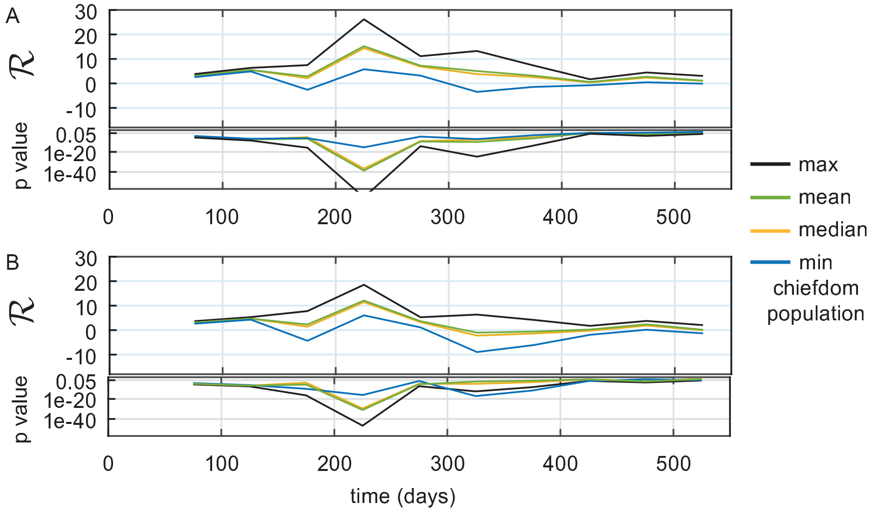

FIG. S8. Effect of chiefdom population on model preference. (A) The likelihood ratio using $(\rho^{\star}, \tau_2^{\star})$ is plotted for different assumptions on how to assign chiefdoms to a sequence case with only known district information. We show how the likelihood ratio timecourse changes depending on choosing the chiefdom in the district according to the maximum, minimum, median, and mean chiefdom population. (B) A similar plot is shown in (A). Here, the gravity model parameters are fixed at $(\rho = 1.6, \tau_2 = 1)$ and $\rho = 1.6$ in the power law model.

I. Observed mean chiefdom size

Virus genomes with unknown chiefdom localization were assigned the median population of the known district. This population assignment was implemented because of three main reasons: i) the confirmed case data shows that the maximum populated chiefdom is usually more than twice as populous as the mean population; ii) the mean and median population approximations provide similar results for the likelihood ratio, seen in Figure S8; and iii) the calculation of a discrete d_{ij} requires a specific chiefdom, which is not possible when using the mean value. Population approximations at the chiefdom level for the virus genome data can be compared to the localization metadata for the confirmed cases[37]. We computed the mean chiefdom population for confirmed cases by district in the three stages of the epidemic, shown in Table S2. We also describe the percentage of total confirmed cases by district in Table S2. The mean chiefdom population in each district changes throughout the epidemic since the set of chiefdoms with confirmed cases varies, except in the Western Area. For Western Urban, there is only one Admin-3 geographic shape in our database. Western Rural does not have Admin-3 level resolution from the metadata for confirmed cases. In stage I of the epidemic, for the two districts with the most confirmed cases, the mean chiefdom population is 93% of the maximum value in Kenema and 54% of the maximum value in Kailahun. In stage I, we found that the virus genome and confirmed case data are consistent indicating that cases appear in chiefdoms with populations closer to the maximum. However, in stage II and III when the virus genomes do not have chiefdom localization, we see that the observed mean population for confirmed cases is typically half the maximum chiefdom population in each district, shown in Table S2.

TABLE S2. Chiefdom population statistics for confirmed cases

	**Percent total cases**			**Mean infected pop./max pop.**		
**Stage**	I	II	III	I	II	III
**District**						
Kailahun	45.7	3.0	0.1	0.54	0.53	0.57
Kenema	38.7	3.3	0.3	0.93	0.66	1
Kono	1.1	3.5	2	0.20	0.42	0.48
Bombali	1.0	15	6	0.63	0.67	0.48
Kambia	0.1	1.9	13	1	0.74	0.77
Koinadugu	0	1.6	0.7	0	0.99	0.95
Port Loko	3.7	14.5	21.8	0.58	0.72	0.84
Tonkolili	0.3	6.6	1.4	0.63	0.54	0.63
Bo	5.0	4.1	0	0.67	0.56	0
Bonthe	0.1	0.1	0	0.98	0.62	0
Moyamba	1.1	2.9	0.8	0.65	0.76	0.80
Pujehun	0.43	0.42	0	0.76	0.66	0
W. Rural	0.9	15.4	11.1	0.27	0.27	0.27
W. Urban	1.7	27.8	42.5	1	1	1

J. Evaluating the likelihood ratio for the three stages

The likelihood ratio, $\mathcal{R}$, is computed for a set of virus genome linkages $\mathcal{S}$. Here, we consider three separate sets of linkages corresponding to the sequenced cases in the three stages of the epidemic. The normalized log-likelihood ratio of a gravity model to a Lévy flight model is: $\mathcal{R}(\rho, \tau_2) = \sum_{\mathcal{S}}[ln(p^G_{ij}) - ln(p^L_{ij})]/\sqrt{N}$, where N is the number of linkages in $\mathcal{S}$. We used the MLE of the parameters for both the gravity and Lévy flight models. In Figure S9, the likelihood is computed for each individual linkage for both models. For each stage, the log-likelihoods at $ln(0.5) = -0.69$ correspond to stationary linkages. Figure S9 illustrates the relative likelihood evaluation between models for each sequence providing insight into the likelihood ratio calculation.

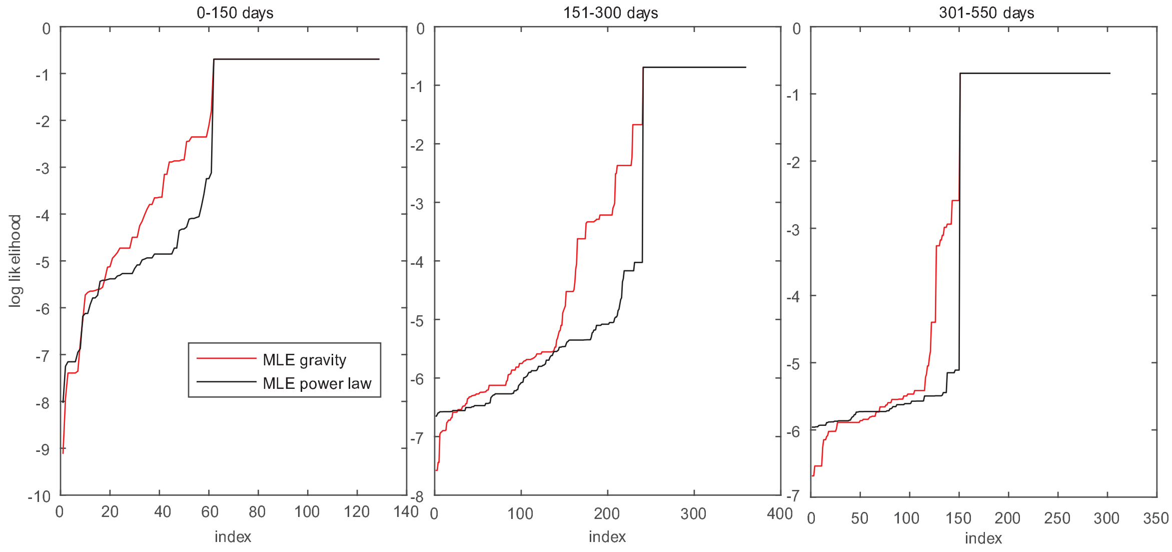

FIG. S9. Log-likelihoods for each transmission linkage for the gravity and Lévy models. The log-likelihood for each individual linkage is plotted for both models. Black lines are for the power law model and red lines are for the gravity model.
